# PGC-1β Induces Susceptibility To Acetaminophen-Driven Acute Liver Failure

**DOI:** 10.1038/s41598-019-53015-6

**Published:** 2019-11-14

**Authors:** Elena Piccinin, Simon Ducheix, Claudia Peres, Maria Arconzo, Maria Carmela Vegliante, Anna Ferretta, Elena Bellafante, Gaetano Villani, Antonio Moschetta

**Affiliations:** 1 Department of Interdisciplinary Medicine, “Aldo Moro” University of Bari, 70124 Bari, Italy; 2 Consorzio Mario Negri Sud, 66030 Santa, Maria Imbaro Italy; 3 INBB, National Institute for Biostuctures and Biosystems, 00136 Rome, Italy; 4 National Cancer Research Center, IRCCS Istituto Tumori Giovanni Paolo II, 70124 Bari, Italy; 5 Department of Basic Medical Sciences, Neurosciences and Sense Organs, “Aldo Moro” University of Bari, 70124 Bari, Italy; 6 Present Address: Institut du thorax, INSERM, CNRS, University of Nantes, Nantes, France

**Keywords:** Hepatotoxicity, Transgenic organisms

## Abstract

Acetaminophen (APAP) is a worldwide commonly used painkiller drug. However, high doses of APAP can lead to acute hepatic failure and, in some cases, death. Previous studies indicated that different factors, including life-style and metabolic diseases, could predispose to the risk of APAP-induced liver failure. However, the molecular process that could favor APAP hepatotoxicity remains understood. Here, we reported that a short-term high fat-enriched diet worsens APAP-induced liver damage, by promoting liver accumulation of lipids that induces the activation of peroxisome proliferator-activated receptor gamma coactivator 1-beta (PGC-1β). Therefore, we challenged mice with hepatic-specific PGC-1β overexpression on a chow diet with a subtoxic dose of APAP and we found that PGC-1β overexpression renders the liver more sensitive to APAP damage, mainly due to intense oxidative stress, finally ending up with liver necrosis and mice death. Overall, our results indicated that during high fat feeding, PGC-1β adversely influences the ability of the liver to overcome APAP toxicity by orchestrating different metabolic pathways that finally lead to fatal outcome.

## Introduction

Overdose of Acetaminophen (APAP) is the most frequent cause of acute and severe liver injury in the Western Countries. APAP is one of the most common analgesic drugs worldwide, safe when used at therapeutic dosage. Nevertheless, its ingestion only 2- or 3-fold over the maximum daily recommended dose can be fatal, due to hepatotoxicity and centrilobular hepatic necrosis^1^. The mechanism of APAP toxicity has been well studied. In normal conditions, APAP is mainly metabolized by glucuronidation and sulfation process. However, during an overdose these pathways become saturated and APAP is mostly metabolized by the Cytochrome P450 CYP2E1 and CYP3A4 isoenzymes into its reactive intermediate NAPQI (N-acetyl-p-benzoquinone imine)^2^. At subtoxic level, NAPQI is detoxified by conjugation with reduced glutathione (GSH), both non-enzymatically and in a reaction catalyzed by glutathione S-transferases. Under APAP overdose conditions, a GSH severe depletion together with a significant increase of NAPQI occurs, leading to mitochondrial dysfunction, ATP depletion, oxidative stress, DNA damage and, finally, cell death.

Even when used at low dose, APAP causes the impairment of mitochondrial respiratory chain, decreasing ATP reservoir and promoting reactive oxygen species accumulation^3^. Together with the reversible mitochondrial dysfunctions, this xenobiotic could exacerbate hepatic fat accretion by inhibiting fatty acids oxidation and intensifying *de novo* lipogenesis, finally inducing steatosis^4,5^. One of the factors that can increase the risk and the severity of APAP-induced liver injury is malnutrition and related metabolic diseases. Indeed, the APAP hepatotoxicity seems to be more frequent in obese or NAFLD (Non-Alcoholic Fatty Liver Disease) patients, even if some studies reported contradictory results, in which obese subjects show similar or decreased risk of APAP-induced hepatic injury compared to non-obese one^6–9^. Most likely, the risk and the severity of APAP toxicity in obese individuals depend on a fragile equilibrium between multiple metabolic processes that can either provide protection or be harmful to the patient itself. Therefore, the identification of pathophysiological molecular factors implied in APAP hepatoxicity is mandatory in order to prevent unintentional hazards to the health of users.

To address this issue, we reported here our results identifying the peroxisome proliferator-activated receptor gamma coactivator 1-beta (PGC-1β) as an important contributor to APAP-induced hepatic failure. The coregulator PGC-1β belongs to the PGC-1s family, whose members play a key role in the control of energy metabolism in many tissues and have been so far recognized as master regulators of mitochondrial biogenesis and antioxidant response^10–13^. Beside its role in oxidative metabolism, in the liver PGC-1β is mainly involved in *de novo* lipogenesis and hepatic triglycerides secretion through very low-density lipoproteins^14,15^. However, despite several studies have been conducted to dissect the role of this coactivator in healthy and pathological conditions, a clear picture of the PGC-1β contribution to liver disease is still missing^16^. By using a gain of function mouse model, we unravel the knot of an intricate scenario where PGC-1β is the mastermind able to orchestrate different hepatocellular processes that increase APAP sensitivity, finally leading to acute hepatic failure.

## Results

### High fat diet fed mice are more prone to APAP hepatotoxicity and mortality

Obesity and related complications arise as a consequence of wrong lifestyle, including malnutrition. Although, usually, several months of high caloric diet are necessary to develop these morbid conditions, short-term exposition to this type of diet is sufficient to induce metabolic changes that can favor liver injury susceptibility.

**Figure 1:**
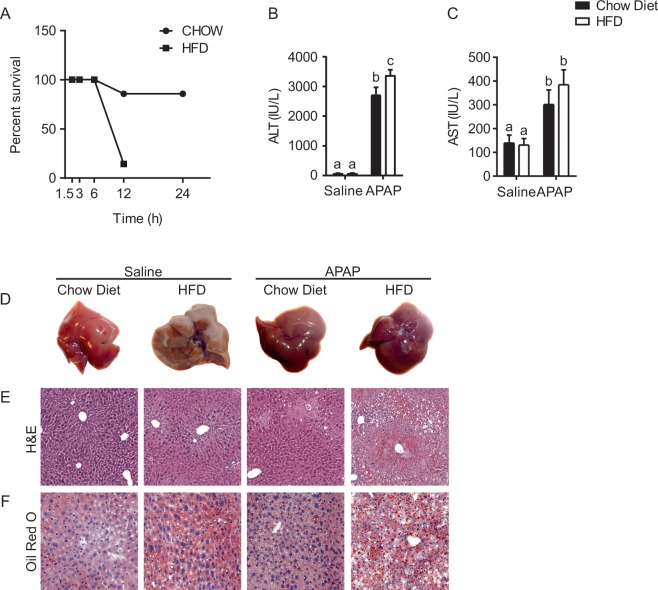
High fat diet fed mice are more prone to APAP hepatotoxicity and mortality. Eight weeks old FVB/N mice fed with HFD or chow diet for 1 month were intraperitoneally injected with either APAP (300 mg/Kg body weight) or equal volume of saline as vehicle control. Survival rate (**A**) of WT mice fed either with high fat or control diet at different time points after APAP injection. ALT (**B**) and AST (**C**) analysis from serum collected 6 hours after injection. Gross morphology (**D**) of livers in mice of indicated treatments and staining of relative liver sections with H&E (**E**), and Oil Red O’ (**F**). Comparison of different groups (n = 6 mice/group) was performed using Two-way ANOVA followed by Bonferroni post-test. Data from groups sharing the same lowercase letters were not significantly different, whereas data from groups with different case letters were significantly different (p < 0.001).

To explore the effects of a short-term high fat diet on APAP-induced liver injury, we subjected 4-weeks old wild type mice to a diet containing 58% fat-derived calories (HFD) for one month. Mice fed with HFD display higher body weight increase compared to control mice (mice fed with Chow Diet, CD) (Supplementary Figure 1A). Thereafter, we intraperitoneally injected the mice with a toxic dose of APAP (300 mg/kg) and we monitored their survival until 24 hours post-injection. The mortality rate in HFD fed mice was much higher compared to CD fed mice throughout the observation period. At 12 hours after APAP injection, the surviving rate of HFD fed mice was less than 10%, whereas over 80% of mice under control diet remained alive (Fig. 1A), suggesting that even a short-term exposition to HFD worsened the APAP-induced hepatotoxicity and mortality.

Correspondingly, HFD-APAP treated mice sacrificed 6 hours later displayed higher levels of both alanine aminotransferase (ALT) and aspartate transaminase (AST) (Fig. 1B,C) compared to CD-APAP treated ones. Gross morphology and histological analysis of the liver revealed a worse phenotype in HFD-APAP-treated mice compared to controls, with clear necrotic areas and massive accumulation of large lipid droplets as indicated by H&E and Oil Red O staining, respectively (Fig. 1D–F).

**Figure 2:**
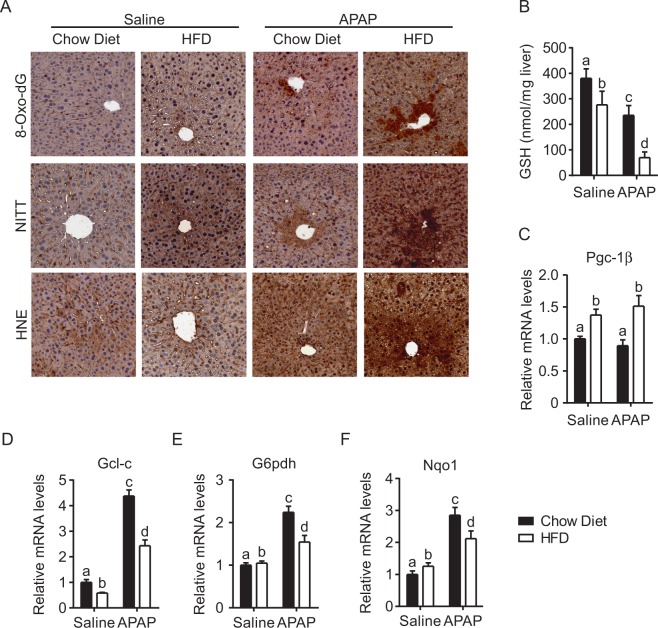
High fat diet fed mice treated with APAP display increase oxidative damage. Eight weeks old FVB/N mice fed with HFD or chow diet for 1 month were intraperitoneally injected with either APAP (300 mg/Kg body weight) or equal volume of saline as vehicle control. Liver tissues were collected 6 hours after injection. (**A**) Staining of the liver sections with 8-Oxo-DG, NITT, and HNE. (**B**) GSH determination on liver tissues. Relative PGC-1β expression (**C**), and of genes related to GSH metabolism, Gcl-c (**D**), G6pdh (**E**) and Nqo1 (**F**) were evaluated by Real Time qPCR. Relative Gene Expression was evaluated in liver specimens from different groups, using TBP as housekeeping gene. Comparison of different groups (n = 6 mice/group) was performed using Two-way ANOVA followed by Bonferroni post-test. Data from groups sharing the same lowercase letters were not significantly different, whereas data from groups with different case letters were significantly different (p < 0.001).

Then, we evaluated GSH and ROS content in liver tissue isolated from CD and HFD mice treated with or without APAP. HFD fed mice injected with saline displayed lower levels of GSH than controls, supporting the idea that fat accumulation conditions *per se* may contribute to deplete GSH reservoir (Fig. 2B). When injected with APAP, HFD fed mice showed three-times less GSH than chow diet fed ones. Notably, hepatic GSH determination after 30 min displayed the same rate of glutathione depletion between the two groups (Supplementary Figure 1B). Consistently, the analysis of ROS damage to DNA, proteins and lipids (8-Oxo-2′-deoxyguanosine, Nitrotyrosine and 4-Hydroxynonenal, respectively) confirmed the presence of a mild oxidative stress in saline injected mice exposed to HFD. APAP administration worsened the hepatic oxidative stress condition, as indicated by the higher accumulation of oxidized derivatives in APAP-treated HFD fed mice compared to CD fed ones (Fig. 2A). Moreover, the expression of antioxidant genes involved in glutathione metabolism were severely downregulated after HFD and APAP challenge. Specifically, the consumption of diet with high fatty acids content negatively affected the quantity of the catalytic subunit of glutamate cysteine ligase (Gcl-c), coding for the rate limiting enzyme of glutathione synthesis, both after saline and APAP injection (Fig. 2D). Also, when challenged with APAP, mice exposed to HFD showed a significantly downregulation of cytoprotective enzymes coding genes glucose 6-phosphate dehydrogenase (G6pdh) and NAD(P)H dehydrogenase 1 (Nqo1) as compared to their CD counterparts (Fig. 2E–F).

Since one of the major regulators of both hepatic lipids and ROS metabolism is the coactivator Pgc-1β^16^, we analyzed its expression in our liver specimens. HFD fed mice display a significative induction of hepatic Pgc-1β expression compared to CD ones, that is preserved after APAP treatment (Fig. 2C, Supplementary Figure 1C). Notably, when sacrificed 3 hours after APAP injection, CD fed mice displayed halved Pgc-1β mRNA levels (Supplementary Figure 1D).

### Hepatic PGC-1β overexpression exacerbates APAP-induced hepatotoxicity and mortality in mice

Since Pgc-1β is one of the master regulators of liver metabolism and given its increased expression observed when mice were challenged with HFD, we decide to decipher whether PGC-1β induction could contribute to the worsening of APAP-induced hepatotoxicity. Previously generated mice overexpressing PGC-1β specifically in the liver, LivPGC-1β, were subjected to a single dose injection of APAP (300 mg/Kg) 8 weeks after birth, together with matched age control mice.

**Figure 3:**
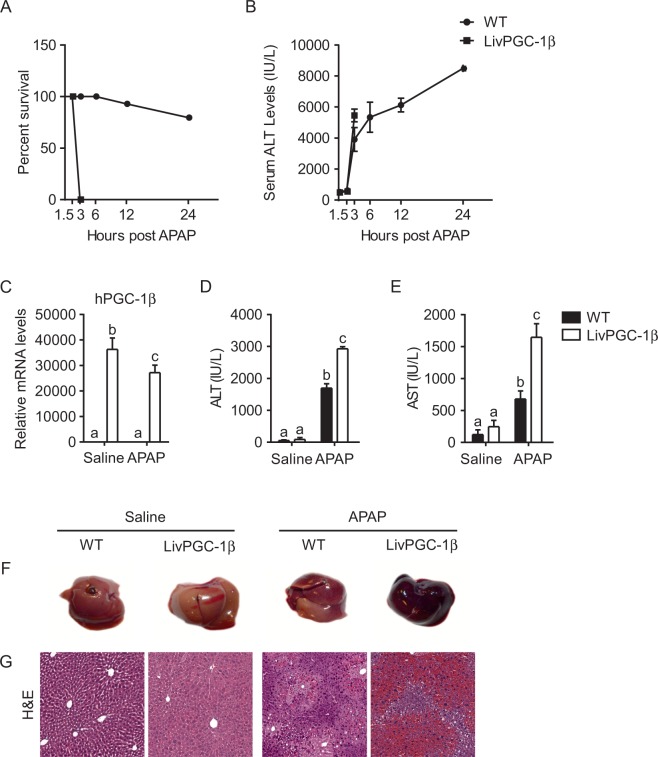
Hepatic PGC-1β overexpression exacerbates APAP-induced hepatotoxicity and mortality in mice. Eight weeks old WT and age matched LivPGC-1β mice were intraperitoneally injected with either APAP (300 mg/Kg body weight) or equal volume of saline as vehicle control and sacrificed. Survival rate (**A**) of either WT and LivPGC-1β at different time points after APAP injection. Serum levels of ALT (**B**) measures at various time point. (**C**) Relative PGC-1β expression evaluated by Real Time qPCR in liver specimens from different groups, using TBP as housekeeping gene. ALT (**D**) and AST (**E**) analysis from serum collected 3 hours after injection. Gross morphology of livers in mice of indicated treatments (**F**) and relative section staining with H&E (**G**). Comparison of different groups (n = 6 mice/group) was performed using Two-way ANOVA followed by Bonferroni post-test. Data from groups sharing the same lowercase letters were not significantly different, whereas data from groups with different case letters were significantly different (p < 0.001).

We monitored the survival of both WT and LivPGC-1β mice for 24 hours after the i.p. injection with 300 mg/kg of APAP. Consistently, we observed a higher mortality rate in transgenic mice compared to WT ones (Fig. 3A). Indeed, three-hours post APAP-injection all the mice overexpressing PGC-1β in the liver died, whereas the survival of WT mice decreased to about 80% only after 24 hours from APAP administration. Accordingly, LivPGC-1β mice displayed significantly higher concentration of serum ALT than control ones (Fig. 3B).

To analyze the factors that may affect LivPGC-1β mortality after APAP treatment, we sacrificed 3 hours post-APAP injection. LivPGC-1β mice displayed high PGC-1β expression in the liver also after APAP challenge (Fig. 3C, Supplementary Figure 2). Serum analysis showed that transaminases levels raised in LivPGC-1β mice treated with APAP, confirming that a more severe hepatic liver failure occurred in transgenic mice compare to WT ones (Fig. 3D,E). Liver gross morphology revealed alterations of hepatic parenchyma of WT mice injected with APAP, whereas LivPGC-1β under the same treatment displayed a necrotic liver (Fig. 3F). These observations were further confirmed by histological analysis, indicating a worse liver appearance in transgenic mice challenged with APAP, characterized by the complete loss of lobular structure (Fig. 3G).

Overall, these data suggest that the overexpression of PGC-1β in the liver renders mice more prone to acetaminophen-induced liver injury.

### Hepatic PGC-1β overexpression markedly increases lipogenic genes even under APAP challenge

**Figure 4:**
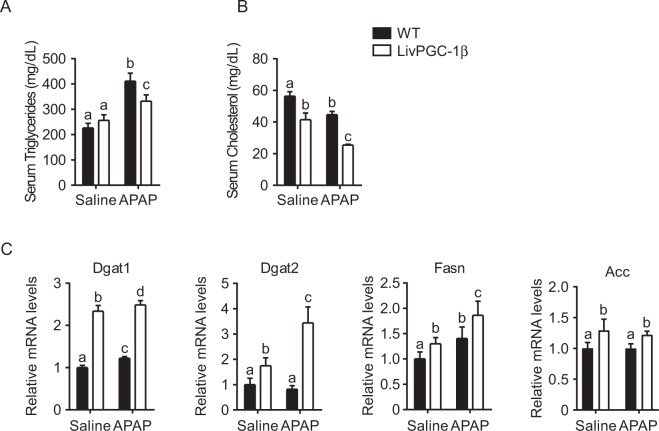
Hepatic PGC-1β overexpression markedly increases lipogenic genes even under APAP challenge. Eight weeks old WT and age matched LivPGC-1β mice were intraperitoneally injected with either APAP (300 mg/Kg body weight) or equal volume of saline as vehicle control. Liver tissues were harvested 3 hours later. Analysis of serum triglycerides (**A**) and cholesterol (**B**) collected from mice. (**C**) Relative expression of genes involved in fatty acids synthesis and excretion. Relative Gene Expression was evaluated by Real Time qPCR in liver specimens from different groups, using TBP as housekeeping gene. Comparison of different groups (n = 6 mice/group) was performed using Two-way ANOVA followed by Bonferroni post-test. Data from groups sharing the same lowercase letters were not significantly different, whereas data from groups with different case letters were significantly different (p < 0.01).

In the liver, PGC-1β plays a major role in the synthesis of new fatty acids and VLDL excretion. To dissect if the hepatic PGC-1β overexpression may heighten APAP response interfering with de novo lipogenesis process, we examined lipid profiles in the four groups. APAP challenge intensified the differences observed in serum lipids profile, being serum triglycerides and cholesterol lower in LivPGC-1β treated mice compared to controls (Fig. 4A,B). Conversely, gene expression analysis displayed increased levels of Fatty Acids Synthase (Fasn) and Acetyl-CoA Carboxylase (Acc) together with the two isoforms of the Diacylglycerol acyltransferase (Dgat1 and Dgat2, respectively) in LivPGC-1β mice compared to controls (Fig. 4C). Thus, the ability of PGC-1β in fostering *de novo* lipogenesis and triglycerides production is further induced when transgenic mice are challenged with APAP, with concomitant lowered excretion of triglycerides and cholesterol.

### Hepatic PGC-1β overexpression increases APAP-induced ROS accumulation due to altered glutathione metabolism

**Figure 5:**
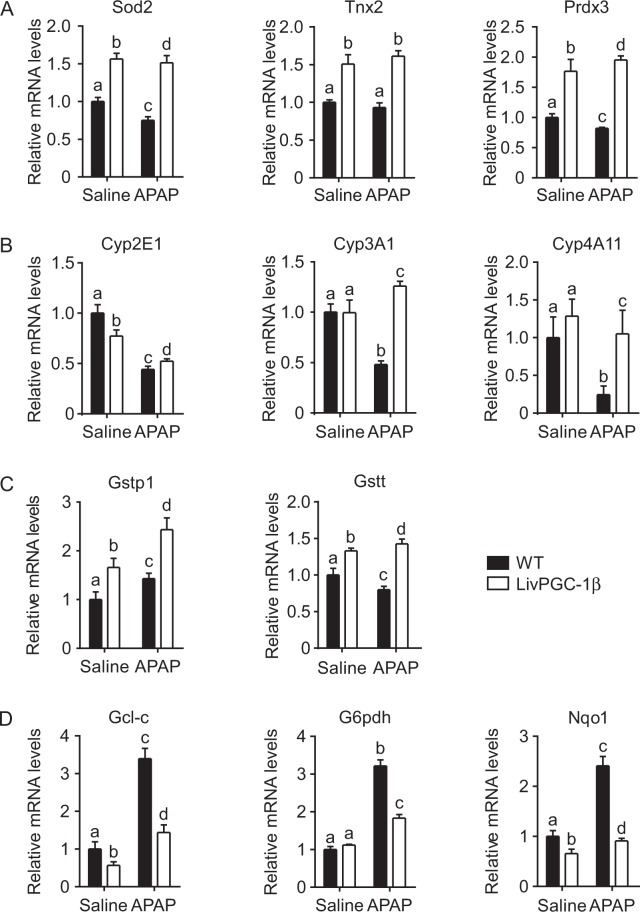
Hepatic PGC-1β overexpression exacerbates APAP-toxic damage due to altered glutathione metabolism. Gene expression analysis on liver specimens isolated from eight weeks old WT and age matched LivPGC-1β mice 3 hours after APAP (300 mg/Kg body weight) or saline treatment. Relative expression of antioxidant genes involved in ROS detoxification (**A**), of different Cytochrome P isoforms involved in APAP bioactivation (**B**), of Glutathione S-transferase (**C**) and of genes implicated in GSH biosynthesis (**D**). Relative Gene Expression was evaluated by Real Time qPCR in liver specimens from different groups, using TBP as housekeeping gene. Comparison of different groups (n = 6 mice/group) was performed using Two-way ANOVA followed by Bonferroni post-test. Data from groups sharing the same lowercase letters were not significantly different, whereas data from groups with different case letters were significantly different (p < 0.01).

**Figure 6:**
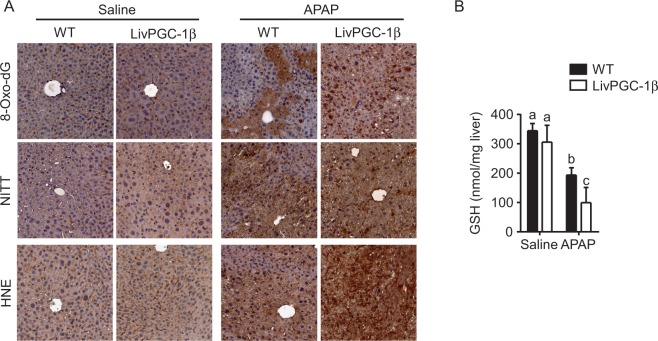
Hepatic PGC-1β overexpression increases APAP-induced ROS byproducts accumulation. Analysis of oxidative stress on liver specimens isolated from eight weeks old WT and age matched LivPGC-1β mice 3 hours after APAP (300 mg/Kg body weight) or saline treatment. 8-Oxo-DG, NITT and HNE staining (**A**) of liver sections isolated from WT and LivPGC-1β mice subjected to different treatments. (**B**) GSH determination from liver tissues. Comparison of different groups (n = 6 mice/group) was performed using Two-way ANOVA followed by Bonferroni post-test. Data from groups sharing the same lowercase letters were not significantly different, whereas data from groups with different case letters were significantly different (p < 0.01).

Lipid accumulation *per se* has been often associated with an increased ROS content. Thus, in the context of acetaminophen hepatoxicity, lipid accretion may aggravate the oxidative status of the liver. However, one of the fundamental roles of PGC-1β relies on the promotion of the antioxidant response, thus limiting the ROS content within a narrow range. Therefore, to understand if PGC-1β was still able to induce ROS scavenger after APAP injection, we analyzed the relative expression of antioxidant genes. LivPGC-1β mice displayed a significative increase of Superoxide dismutase 2 (Sod2), Thioredoxin 2 (Txn2) and Peroxiredoxin 3 (Prdx3) mRNA levels, that was still preserved after APAP injection. On the contrary, WT mice showed a small but significant reduction of these mRNA levels when challenged with the drug (Fig. 5A). ROS scavengers induction lowered the oxidative stress in LivPGC-1β mice compared to controls after saline injection. Indeed, the evaluation of ROS-mediated lipid damage exhibited prominent HNE accumulation in WT mice, with clear marked nuclei, possible due to HNE covalent adducts with nucleophilic functional groups in nucleic acids^17^. Surprisingly, this protective effect was completely lost after treatment with APAP, with the transgenic mice displaying a remarkable HNE deposition (Fig. 6A). The greater extent of oxidative damage in LivPGC-1β mice treated with APAP compared to WT ones is also indicated by the 8-Oxo-2′-deoxyguanosine and the Nitrotyrosine staining, markers of ROS-byproducts affecting DNA and proteins, respectively (Fig. 6A).

In line with these observations, we found halved concentration of hepatic GSH in APAP-treated LivPGC-1β mice compared to control ones (Fig. 6B). To explore whether the loss of GSH reservoir, the major scavenger system against oxidative stress caused by APAP, was attributable to an increased acetaminophen metabolization or to a decreased glutathione synthesis, we investigated the relative expression of the main genes involved in these processes. The analysis of different isoforms of cytochrome P450 (Cyp2E1, Cyp3A1 and Cyp4A10), involved in bioactivation of APAP into NAPQI, revealed a significant higher expression in transgenic mice challenged with APAP compared to control ones (Fig. 5B). Remarkably, the overexpression of PGC-1β fostered the increase of Glutathione S-transferases (Gstt, Gstp), involved in the detoxification of NAPQI by conjugation with GSH (Fig. 5C). Therefore, it is possible that overexpression of PGC-1β not only induced a faster metabolization of the drug, with a concomitant accumulation of highly reactive intermediates, but also contributed to a rapid GSH depletion by Glutathione S-transferases.

Nonetheless, after APAP treatment the expression of antioxidant genes with a prominent role in the glutathione metabolism (Gcl-c, G6pdh and Nqo1) raised in WT mice, while only modestly increased in LivPGC-1β littermates (Fig. 5D). Particularly, the mRNA levels of Gcl-c, the first-rate limiting enzyme of glutathione synthesis, were already low in LivPGC-1β injected with saline, suggesting a possible negative correlation between coactivator and enzyme expression. Overall, these data suggested that mice overexpressing PGC-1β display deleterious effects when challenged with APAP, since they failed in the induction of genes involved in GSH synthesis, thereby limiting the GSH reservoir to contrast acetaminophen overdose.

## Discussion

Acetaminophen (APAP) is a painkiller drug, extensively used worldwide. However, high dosage of APAP induces rapid necrosis of hepatic tissue, that could end up with ill-fated outcome and patients’ death. Although it is well known that these adverse events of acetaminophen use are mostly attributable to the depletion of GSH reservoir^18^, it is still to be defined why some people are more sensitive to the APAP side effects and how to predict the fatal end.

Recent observations pointed out that obesity and related metabolic diseases may affect APAP liver injury. However, both clinical investigations and experimental studies reported contradictory results, in which obese and NAFLD subjects displayed either higher or lower hepatotoxicity after APAP administration^6,9,19–21^. Thus, an unequivocal mechanism whereby APAP could be more toxic in obese condition and related disorders is still to be determined.

It is well known that malnutrition plays a fundamental role in overweight and obesity. Diets containing an excess or imbalance of energy can lead to overnutrition and fat accumulation, that predispose to body weight increase. Here, we showed that a short but extreme oversupply of fatty acids with the diet is able to worsen APAP administration. Mice rapidly died due to a drastic lowering of GSH supply, possibly ascribable to the decreased expression levels of several enzymes involved in glutathione biosynthesis. Interestingly, we found high hepatic expression of PGC-1β in mice that received high fat diet independent of drug treatment, whereas APAP *per se* halved the mRNA levels of PGC-1β in a chow diet background, thus implying a kind of protective mechanism exerted in normal conditions.

The coactivator PGC-1β belongs to the family of PGC-1s coactivators, generally considered as master regulators of mitochondrial biogenesis and oxidative metabolism as well as of antioxidant response^22^. In the liver, PGC-1β has been shown to play a key role in the *de novo* lipogenesis and triglycerides metabolism^12,14,15^. Former experiments on LivPGC-1β mice indicate that hepatic overexpression of PGC-1β may differently impact on metabolic pathways. Indeed, selective activation of PGC-1β within the hepatocytes protects the liver from lipids overload and progression to fibrosis, due to its ability to induce mitochondrial oxidative phosphorylation, fatty acids β-oxidation and citrate cycle, while decreasing oxidative stress and promoting triglycerides excretion in the bloodstream^15^. On a different angle, when subjected to DEN-induced hepatocarcinogenesis, overexpression of PGC-1β renders mice more prone to hepatocarcinoma development possibly by enhancing the expression of genes involved in fatty acids and triglycerides synthesis as well as by limiting ROS accumulation via increased antioxidant response^12^.

Previous studies demonstrated that the other member of the PGC-1’s family, PGC-1α, protects the liver from acetaminophen-induced liver injury by inducing the expression of Nrf2-regulated anti-oxidant genes^23^. Moreover, the induction of PGC-1α after APAP hepatotoxicity promotes mitochondrial biogenesis that finally attenuates the injury and stimulates liver regeneration^24^. Of notice, the hepatic overexpression of PGC-1β leads to an acute liver failure after subtoxic dose of APAP, inducing mice death three hours post-injection. These evidences highlighted once more the divergent role of the two coactivators in the liver, with PGC-1α being more involved in catabolic pathways and PGC-1β inducing anabolic processes, such as the synthesis of new fatty acids.

If APAP injection is able to stimulates fatty acids accretion *per se*^4^, the overexpression of PGC-1β boosts the expression of genes involved in *de novo* lipogenesis and triglycerides synthesis, thus contributing to the impairment of normal liver functions. Moreover, PGC-1β overexpression induces higher hepatotoxicity also exacerbating the oxidative stress that finally leads to liver failure and mice death.

Hepatic oxidative stress is the principal mediator of APAP induced liver injury. N-Acetyl-p-benzoquinone imine (NAPQI), the toxic metabolite of APAP, besides its intrinsic ability to form aspecific chemical adducts with proteins, promotes the excessive accumulation of ROS mostly depleting the GSH pool that it is required for its detoxification. This, in turn, can cause alterations of mitochondria and nucleic acids, ceasing the ATP production and contributing to liver necrosis^1,25,26^. We demonstrated that PGC-1β overexpression leads to an increase of cytochrome P450 isoforms (Cyp) which results in the intensification of NAPQI production, with consequent rising of the oxidative stress. Beside NAPQI, Cyp can also directly increase oxidative stress by consuming NADPH during their catalytic cycle.

At the same time, PGC-1β failed to induce the expression of enzymes involved in glutathione biosynthesis. The glutamate-cysteine ligase catalytic subunit (Gcl-c), the main controlling step of GSH synthesis is less expressed in mice overexpressing PGC-1β at basal conditions. Moreover, whereas its mRNA levels rapidly rise in WT mice after APAP injection, in LivPGC-1β we detected just a feeble increase of Gcl-c, probably not sufficient to enrich the GSH supply useful to limit APAP damage. Surprisingly, one of the inducers of Gcl-c expression is 4-Hydroxynonenal (HNE), the major end product of lipid peroxidation^27,28^. HNE covalently binds to functional groups of proteins, nucleic acids and membrane lipids, driving to serious cell damage, and, ultimately, cell death^17^. In mice with hepatic overexpression of PGC-1β, HNE amount is kept low in basal conditions, thus contributing to the reduced expression levels of Gcl-c observed. Conversely, after APAP injection in hepatic specific PGC-1β overexpressing mice, the high expression of primary ROS scavengers is not able to counteract the hepatic oxidative stress induced by APAP, due to the collapse of GSH reservoir caused by the lowered Gcl-c expression and by the massive utilization of GSH for NAPQI detoxification performed by Glutathione S-Transferases.

Despite the enhanced HNE staining observed in our samples, we have to highlight on one hand that lipid peroxidation *per se* is not always recognized as relevant in APAP hepatotoxicity, and its contribution to liver injury after acetaminophen exposure is still debated^3,29–32^. Also, it may be possible that the increased HNE staining is a specific consequence of the mouse strain used in our experiments, given the fact that FVBN mouse strain might be more sensitive than C57BL/6 J one^33,34^. However, it is also plausible that the enhanced lipid peroxidation observed is the resultant of a combined effect of APAP and predisposition to liver steatosis and its sequelae that once more contribute to liver failure^35^. Thereby, even if lipid peroxidation *per se* is insufficient to cause cell death, it may be considered as evidence for oxidative stress that contribute to liver injury. Indeed, when hepatic PGC-1β levels are high, mice liver appeared intensely affected by oxidative stress, as depicted by extensive accumulation of ROS byproducts (8-Oxo-DG, NITT and HNE), that is suggestive of the prelude to hepatic necrosis and death.

Notably, two of the most important transcription factors coactivated by PGC-1β are Nrf2 (Nuclear Factor E2-Related Factor 2) and LXRs (Liver X Receptors)^16^. Nrf2 is mainly implicated in antioxidant response, whereas LXR controls lipid metabolism^36,37^. Though, recently it has been pointed out that the molecular pathways controlled by these two transcription factors not only are highly overlapping, with Nrf2 able to modulate genes involved in lipid metabolism and LXR involved in the promotion of glutathione transferase, but they are also mutually interconnected^38–40^. In the present study, high level of PGC-1β induced the expression of genes involved in lipid metabolism and detoxification, but failed to promote the expression of Nrf2 regulated genes involved in glutathione synthesis. Lastly, it has been described that hepatic injury caused by APAP is lower in mice with elevated expression of Nrf2 and in those with constitutive activation of LXR^39,41^. Therefore, it is plausible that the effect of PGC-1β overexpression we observed did not rely only on these two transcription factors, but that it was mediated also by other factors, that finally contributed to the deleterious outcome.

Overall, in the present study we highlight that short-term consumption of diets enriched in fatty acids may worsen the risk of hepatic failure due to APAP consumption. Lipidic metabolites or intermediate ligands can be able to activate PGC-1β, mimicking our transgenic mouse model, thus contributing to exacerbate the risk of APAP hepatotoxicity. Unfortunately, the impossibility to study the direct effect on humans, due to obvious reasons, is one of the major limitations of this study. However, a healthy life style, characterized by proper consumption of lipids and carbohydrates that would limit PGC-1β induction, might be a preventive strategy to reduce APAP induced liver damage in human. Our data depict an interesting scenario, in which coactivator PGC-1β is able to increase APAP sensitivity, finally leading to acute hepatic failure.

## Materials and Methods

### Study design

This study was firstly designed to test the hypothesis that the administration of a diets enriched in fatty acids for a short period of time has the potential to worsen APAP-induced liver injury. Since we showed that HFD consumption induces PGC-1β expression, and shortly after injection, APAP treatment halved PGC-1β levels, to validate the PGC-1β as a putative biomarker we examined the effect of APAP treatment on hepatic-specific transgenic mouse model. We demonstrated that PGC-1β limits glutathione production and negatively affect oxidative stress, finally resulting in a severe liver damage. Sample sizes and P values are indicated in the text, figure legends, or figures. The sample sizes of the experiments were selected on the basis of previous experience. Data reported included all samples analyzed. No samples or data were excluded after analysis. Experiments were carried out in an unblinded fashion except for analyses of immunohistochemistry images.

### Animal studies

All animals were housed in a controlled environment with 12 hours light and dark cycles, with free access to food and water. For high fat feeding, four weeks-old wild type (WT) FVBN male mice were switched on diet containing 58% fat-derived calorie (D12331, Research Diets) for one month. A total of four groups of 6/7 male mice each has been tested. Mice were randomized prior treatment with the diet and then allocated in different cages (3/4 mice per cage).

Male LivPGC-1β mice^15^ and wild type (WT) controls in FVBN background were used for study the effect of PGC-1β overexpression. A total of four groups of 6/7 male mice each has been tested. Mice were randomized at 4 weeks of age and then allocated in different cages (3/4 mice per cage).

To induce APAP hepatotoxicity, 8 weeks-old animals were fasted for 16 hours before the experiments. An APAP solution freshly-prepared by dissolving with heating acetaminophen (Sigma-Aldrich) in saline solution was administrated into mice by intraperitoneal injection at 300 mg/Kg. Saline solution (0.90% w/v of NaCl) was used as control. The experimental protocol was approved by the Ethical Committee of the Consorzio Mario Negri Sud and also was certified by the Italian Ministry of Health according to internationally accepted guidelines and regulation for the animal care.

### RNA extraction and quantitative real-time PCR analysis

All the tissues were harvested at indicated time points after APAP injection. Liver were snapped freezing under liquid nitrogen and homogenized in QIAzol reagent (Qiagen) using Tissue Lyser (Qiagen). Total RNA was isolated by following the manufacture’s instruction. To avoid possible DNA contaminations, RNA was treated with DNase (ThermoScientific). cDNA was synthesized retro-transcribing 4 μg of total RNA in a total volume of 400 μl using High Capacity DNA Kit (TehrmoScientific) in accordance to the manufacture’s instruction. Real Time qPCR was performed using SybrGreen Master Mix (ThermoScientific) with various sets of primers for specific genes. The PCR reactions were performed on the Studio Quant5 (ThermoScientific). Relative gene expression levels were analyzed using the ΔΔCt method, using TBP as housekeeping gene.

### GSH assay

For GSH assay *in vivo*, liver specimens were collected at different time points as indicated. Detection of GSH abundance was performed using Glutathione Assay kit (Sigma-Aldrich) following the manufacturer’s instructions. For GSH determination 30 minutes after APAP injection, liver tissue (50 mg) was homogenized with 20 volumes of 5% 5-Sulfosalicylic Acid Dihydrate (500 µL) (Sigma-Aldrich) and centrifuged at 12,000 g for 10 minutes at 4 °C. Total glutathione levels were measured from the supernatants using a glutathione detection kit according to the manufacturer’s instructions (Enzo Life Sciences)^42^.

### Serum analysis

At the time of sacrifice, blood samples were collected in lithium-heparin collection tubes and subsequently centrifuged to obtain serum. Serum levels of ALT and AST were measured using a colorimetric kit (BioQuant, Heidelberg, Germany) according to manufacturer’s instructions.

### Histological and immunohistochemical analysis

Tissue specimens were fixed in 10% formalin for 12 to 24 h, dehydrated, and paraffin embedded. Liver sections were stained with H&E following standard protocols. For immunohistochemistry, sections were subjected to antigen retrieval by boiling the slides in sodium citrate pH 6 (Sigma Aldrich) for 15 min and then permeabilized in phosphate-buffered saline (PBS) with 0.25% TritonX-100 for 5 min. Subsequently, after 10 min incubation at room temperature in protein blocking solution (Dako, Glostrup, Denmark), sections were incubated at 4 °C for 48 hours with the anti-8-Hydroxyguanosine antibody (LifeSpan Bioscience Inc, Seattle, Washington, USA). Sections were washed in PBS for 15 min and incubated for 25 min at room temperature with DAKO real EnVision detection system Peroxidase/DAB + (Dako) according to manufacturer’s instruction. Coverslips were mounted with Permount and evaluated under a light microscope. To evaluate lipid accumulation, serial 4.5 μm cryosections from liver specimens embedded in OCT compound (Tissue-Tek Sakura, Torrance, CA) were stained with Oil Red O (Sigma-Aldrich) and hematoxylin to counterstain nuclei.

### Western blot analysis

Total liver protein lysates were separates on 10% and 12% SDS–polyacrylamide gel and transferred on nitrocellulose membrane. Membranes were blocked with 5% BSA in TBS–0.01% Tween 20 and probed with specific antibodies against PGC-1β (ab176328) and GCL-c (ab53179) purchased from Abcam. Nuclear encoded β-actin (Abcam) were used as loading control. Membranes were finally incubated with HRP conjugated secondary antibodies and developed with a chemiluminescent reagent (Biorad, California, USA).

### Statistical analysis

The results are expressed as mean ± SEM. All the statistical analyses were performed using GraphPad Prism software (v5.0; GraphPad Software Inc). Comparisons of two groups were performed using Mann-Whitney U test. Comparison of four groups were performed using two-way ANOVA followed by Bonferroni post-test. At least p-value < 0.05 was considered statistically significant.

## Supplementary information

Supplementary Material
