# Supplementary material for: PGC-1β Induces Susceptibility To Acetaminophen-Driven Acute Liver Failure
Source: Sci Rep. 2019 Nov 14;9:16821. doi: 10.1038/s41598-019-53015-6 (PMC6856160; doi:10.1038/s41598-019-53015-6)
Supplement: Supplementary file 1 — Supplementary Material [file 41598_2019_53015_MOESM1_ESM.docx]

PGC-1β Induces Susceptibility To Acetaminophen-Driven Acute Liver Failure

Supplementary Material

Elena Piccinin^1,2^, Simon Ducheix^3†^, Claudia Peres^1,3^, Maria Arconzo^3^, Maria Carmela Vegliante^4^, Anna Ferretta^5^, Elena Bellafante^2^, Gaetano Villani^5^, Antonio Moschetta^1.4^*

^1^Department of Interdisciplinary Medicine, “Aldo Moro” University of Bari, 70124 Bari, Italy;

^2^Consorzio Mario Negri Sud, 66030 Santa Maria Imbaro, Italy;

^3^INBB, National Institute for Biostuctures and Biosystems, 00136 Rome, Italy;

^4^National Cancer Research Center, IRCCS Istituto Tumori Giovanni Paolo II, 70124 Bari, Italy;

^5^Department of Basic Medical Sciences, Neurosciences and Sense Organs, “Aldo Moro” University of Bari, 70124 Bari, Italy.

^†^*Present Address:* Institut du thorax, INSERM, CNRS, University of Nantes, Nantes, France

***To whom correspondence should be addressed:** Antonio Moschetta, MD PhD, Clinica Medica “Cesare Frugoni”, Department of Interdisciplinary Medicine, University of Bari “Aldo Moro”, Piazza Giulio Cesare 11, 70124 Bari; Phone: +39 0805593262; Fax: +39 0805555388; E-mail: [antonio.moschetta@uniba.it](mailto:antonio.moschetta@uniba.it)

**Supplementary Figure 1**

**
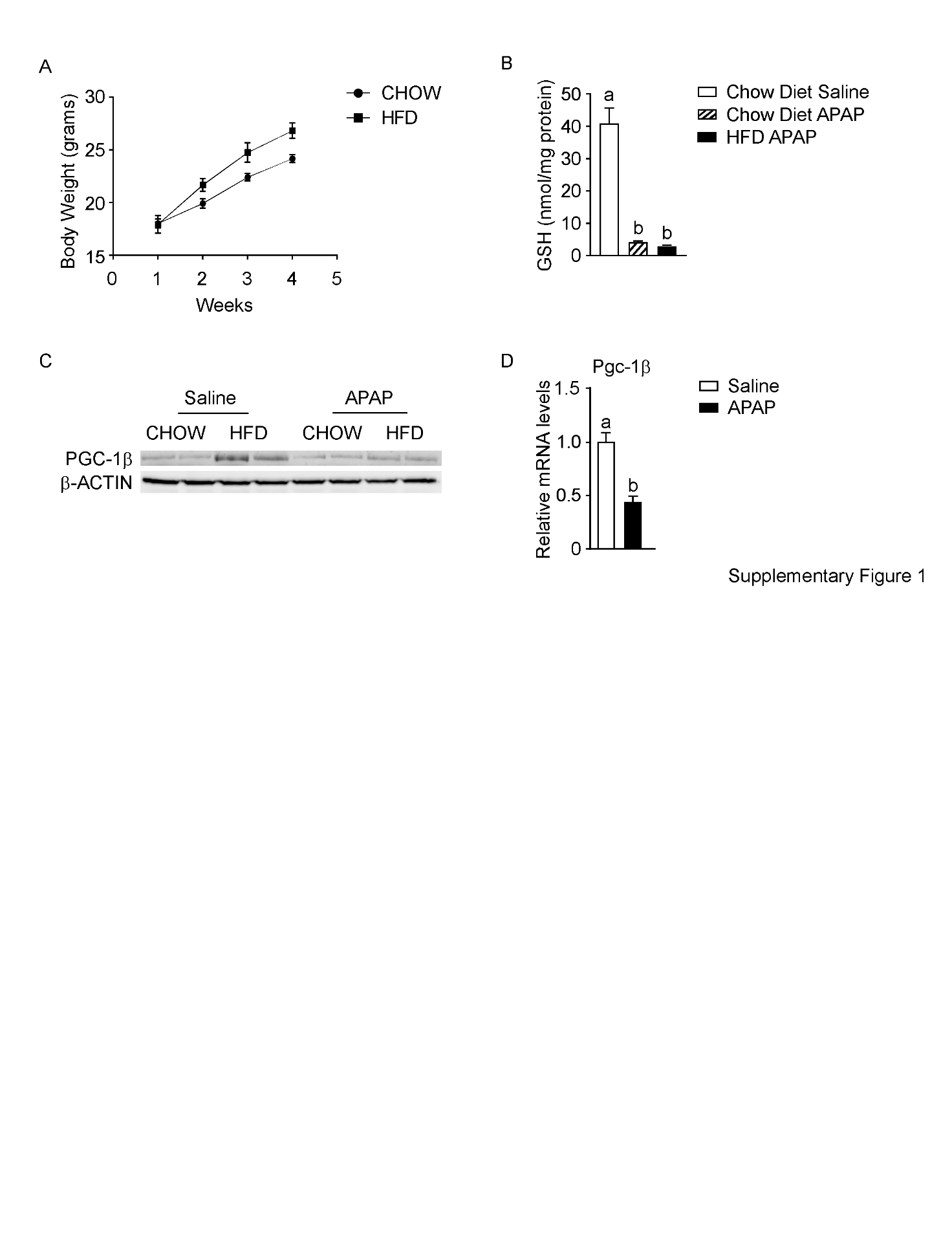
**

**Supplementary Figure 1. High fat diet fed mice display Pgc-1β increased levels, which decreased after APAP administration.** Eight weeks old FVB/N mice fed with either chow or high fat diet (HFD) for 1 month, and then intraperitoneally injected with either APAP (300 mg/Kg body weight) or equal volume of saline as vehicle control. **(A)** Body weight gain of both chow diet and HFD fed mice. **(B)** GSH determination on liver tissue 30 minutes after APAP injection. Comparison of different groups was performed using Kruskal-Wallis test. **(C)** Western Blot analysis of protein lysates obtained from liver tissue of FVB/N mice subjected to different treatments. β-Actin was used as loading control. All samples were executed simultaneously in the same Western blot. PGC-1β and β-actin clearly delineated with white space to show the relevant band. **(D)** Relative expression of PGC-1β in the liver of mice fed with chow diet, after 3 hours of treatment with either saline or APAP. Relative Gene Expression was evaluated by Real Time qPCR in liver specimens from different groups, using TBP as housekeeping gene. Comparison of different groups (n=6 mice/group) was performed using Mann-Whitney U test. Data from groups sharing the same lowercase letters were not significantly different, whereas data from groups with different case letters were significantly different (P<0.01).

**Supplementary Figure 2**

**
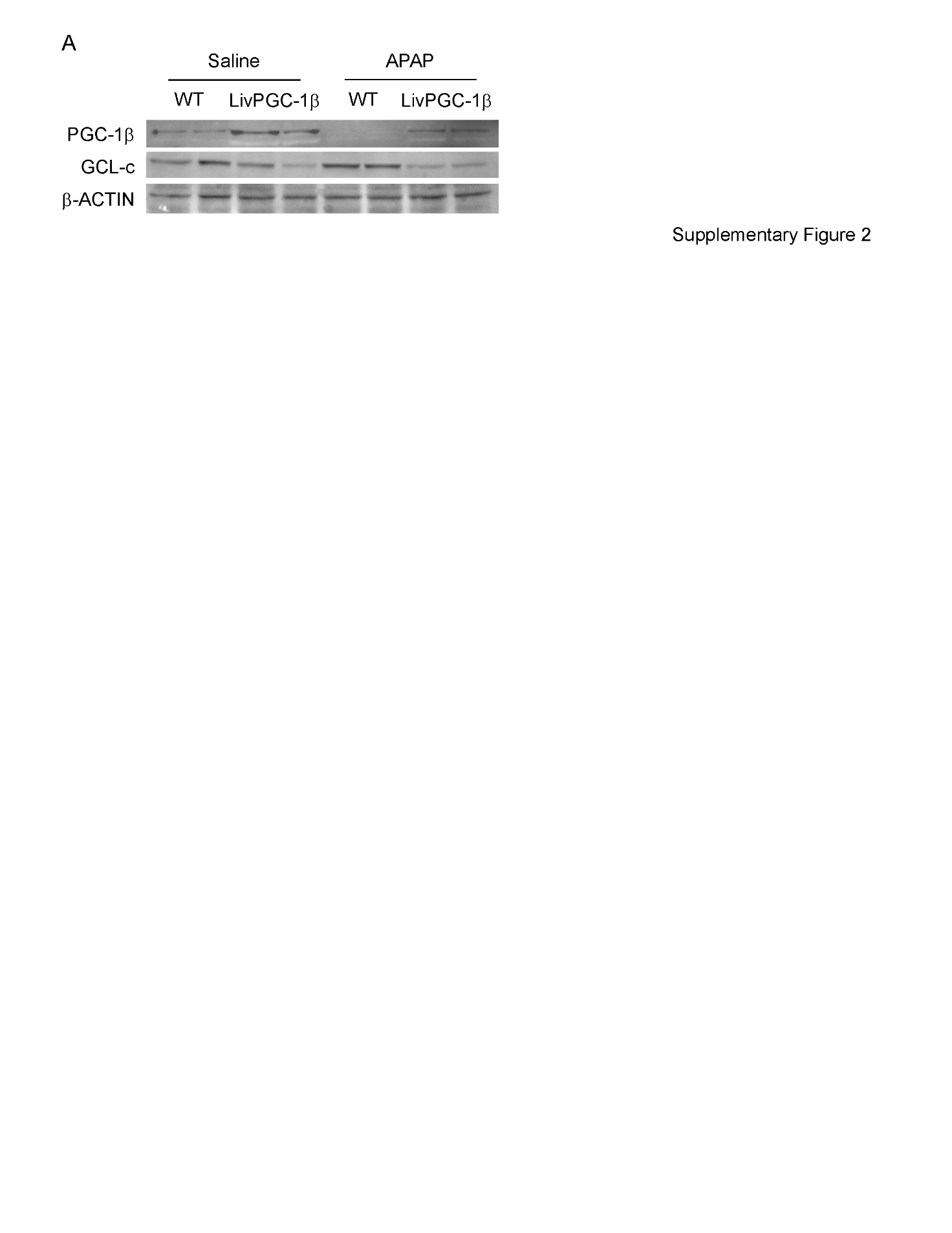
Supplementary Figure 2. Hepatic PGC-1β overexpression limits glutathione synthesis.** Eight weeks old WT and age matched LivPGC-1β mice were intraperitoneally injected with either APAP (300 mg/Kg body weight) or equal volume of saline as vehicle control. Liver tissues were harvested 3 hours later. **(A)** Protein abundance of PGC-1β and Gcl-c determined by Western Blot analysis. β-Actin was used as loading control. All samples were executed simultaneously in the same Western blot. PGC-1β, Gcl-c and β-actin clearly delineated with white space to show the relevant band.
